# Preventing and Managing Vascular Complications in Adolescents With Type 1 Diabetes

**DOI:** 10.1155/jdr/2449658

**Published:** 2025-12-29

**Authors:** Beatrice Prampolini, Cansu Ceren Eryılmaz, M. Loredana Marcovecchio

**Affiliations:** ^1^ Department of Medical and Surgical Sciences for Mothers, Children and Adults-Post-Graduate School of Pediatrics, University of Modena and Reggio Emilia, Modena, Italy, unimore.it; ^2^ Department of Child Health and Disease, Istanbul University Faculty of Medicine, Istanbul University, Istanbul, Turkey, istanbul.edu.tr; ^3^ Department of Paediatrics, University of Cambridge and Cambridge University Hospitals NHS Foundation Trust, Cambridge, UK

**Keywords:** adolescents, prevention, screening, treatment, Type 1 diabetes, vascular complications, youth

## Abstract

The morbidity and mortality associated with Type 1 diabetes are primarily linked to vascular complications. While overt clinical manifestations of microvascular complications and cardiovascular disease predominantly occur during adulthood, a noteworthy proportion of adolescents with Type 1 diabetes have one or more risk factors for the development of vascular complications. In addition, early subclinical stages of vascular complications can be detected already in youth with diabetes. Intensive diabetes management and targeted screening strategies can effectively delay or halt the progression of these complications. Preventative strategies should focus on three main principles: achieving optimal glycemic targets, preventing and targeting cardiometabolic risk factors, and implementing early screening programs. For managing complications, a comprehensive approach is essential, combining insulin therapy to optimize glycemic targets, motivational lifestyle interventions, and timely pharmacological treatments. This review summarizes the epidemiology and risk factors for vascular complications in youth with Type 1 diabetes and emphasizes the importance of early detection and prevention, and effective treatment strategies.

## 1. Introduction

**Figure 1:**
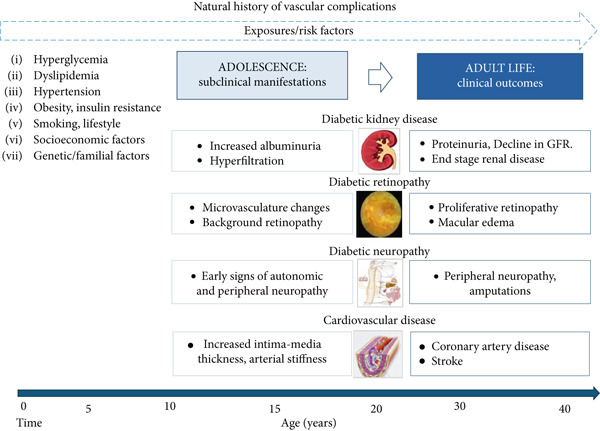
Progression of vascular complications in the lifetime of a person diagnosed with Type 1 diabetes during childhood/adolescence and key risk factors.

Type 1 diabetes is linked to a reduced quality of life, increased morbidity and mortality, and significant global healthcare costs [1]. The long‐term vascular complications associated with Type 1 diabetes primarily contribute to these outcomes. While clinical manifestations of vascular complications typically emerge later in life, many adolescents with Type 1 diabetes already exhibit one or more risk factors and early subclinical signs of vascular damage [2, 3] (Figure 1).

Intensive diabetes management strategies and screening programs tailored to the developmental needs of youth can prevent or delay the onset of complications [4].

This review provides a comprehensive overview of the epidemiology and risk factors associated with vascular complications in young people with Type 1 diabetes. It highlights the critical importance of screening for the early detection of these complications and related risk factors. Additionally, the review emphasizes the importance of early prevention strategies and the implementation of effective treatment approaches to mitigate long‐term health impacts.

The review is based on evidence published primarily in the last 15 years.

## 2. Epidemiology of Type 1 Diabetes and its Complications

According to the latest published data, as of 2025, an estimated 9.5 million people were living with Type 1 diabetes, of whom 1.8 million were younger than 20 years old [1]. The estimated annual incidence of new cases was 503,000, with notable geographic variations. The lowest incidence rates were observed in the Western Pacific and East Asia regions, while the highest rates were reported in North America. Among newly diagnosed cases, 22.3% occurred in individuals under the age of 20 [1]. Furthermore, the number of youths with Type 1 diabetes is projected to increase over the next two decades, particularly in low‐income and low‐and middle‐income countries [5, 6]. These findings are particularly concerning because of the increased burden associated with an early onset of Type 1 diabetes and the longer exposure to hyperglycemia and other cardiometabolic risk factors contributing to vascular complications [7].

Substantial progress in diabetes care has improved glycemic outcomes and reduced cardiometabolic risk factors; however, mortality rates remain high. The disparity in life expectancy between people living with Type 1 diabetes and their peers without diabetes is greater for those diagnosed before the age of 20 years than for those with a diagnosis later in life [7]. The latest global estimates show that a 10‐year‐old child with Type 1 diabetes experiences an average gap in life expectancy of 24 years compared with their peers without diabetes. This gap varies widely based on social and economic conditions, ranging from 46 years in low‐income to 11 years in high‐income countries [5].

The burden associated with diabetes is mainly due to the development of vascular disease, which includes microvascular complications, primarily represented by diabetic retinopathy (DR), diabetic kidney disease (DKD), diabetic neuropathy, and cardiovascular disease (CVD) [8]. Although overt clinical manifestations of vascular complications are typically observed during adulthood, their incidence represents the result of a long subclinical disease process, and early subclinical manifestations can occur during childhood and adolescence [8] (Figure 1).

Data on the prevalence and incidence of vascular complications in youth with Type 1 diabetes are derived from several observational studies conducted in different countries. These studies often involve populations that vary in age, ethnicity, sociodemographic factors, as well as in the methods used to detect complications, which can lead to different results and make comparisons challenging.

Between 2002 and 2015, Dabelea et al. conducted a large observational study in the US to evaluate the prevalence of vascular complications and associated risk factors among young people diagnosed with Type 1 diabetes before the age of 20 years. At a mean age of 21 years and diabetes duration of 7.9 years, the reported prevalence rates were 5.8% for DKD, 5.6% for DR, and 8.5% for peripheral neuropathy. Furthermore, 11.6% of the study population showed increased arterial stiffness, and 10.1% developed hypertension. Overall, 32% of the cohort exhibited at least one diabetes‐related complication [3]. Eppens and colleagues investigated the prevalence of vascular complications in youth from New South Wales and Australia, and reported a prevalence of 20% for DR, 6% for moderately and severely increased albuminuria; 28% for hypertension, 27% for peripheral neuropathy, and 61% for autonomic neuropathy [9]. Cho and colleagues examined the trends in the prevalence of microvascular complications in Australian adolescents aged 11–17 years with Type 1 diabetes from 1990 to 2006. A prevalence of DR of 7% was documented in 2006 compared with 16% in 1990, with a stable prevalence for moderate and severe increases in albuminuria (21% and 3.7%, respectively). In contrast, peripheral neuropathy was observed in 28% of cases in 2006 compared with 14% in 1990 [10].

Concerning data from low‐income settings underscore the global burden of early complications. A recent study by Msanga and colleagues, enrolling children and adolescents aged 5–19 years with diabetes from Tanzania, showed prevalences of 32.9% for DKD, 10.3% for DR, 13.6% for neuropathy, and 41.9% for at least one microvascular complication. Of those presenting with complications, a total of 26% had two complications, and 4.6% had three [11]. However, the cross‐sectional design limits causal inference, and the diagnostic tools—particularly for DKD and neuropathy—were less sensitive than recommended standards. More data on the epidemiology of complications from low‐resource countries are needed.

Even though young people with diabetes predominantly exhibit early manifestations of vascular complications, major adverse clinical events have also been documented. In youths enrolled in the SEARCH for Diabetes in Youth and the Treatment Options for Type 2 Diabetes in Adolescents and Youth (TODAY) studies, incidence rates (per 10,000 person‐years) among those with Type 1 diabetes were 10.9 for ophthalmologic events, 11.1 for neuropathy, 3.1 for cardiac events, 3.1 for peripheral vascular disease, 1.6 for cerebrovascular events, and 15.6 for gastrointestinal complications [12]. These findings align with the long‐term outcomes of the Diabetes Control and Complications Trial (DCCT) and Epidemiology of Diabetes Interventions and Complications (EDIC) studies, which enrolled participants aged 13–39 years, including 195 adolescents (13–17 years). After 30 years of follow‐up, the cumulative incidence reached 33.4% for proliferative DR (PDR), 36.6% for clinically significant macular edema (CSME), 14.1% for reduced eGFR, 3.2% for amputation, 17.1% for CVD, and 10.4% for mortality; notably, only 44% of participants remained free of complications [13].

Complementary evidence from the Adolescent Type 1 Diabetes cardiorenal Intervention Trial (AdDIT) studies further underscores the early burden of complications: after a median follow‐up of 3.2 years, 12% of the adolescents with Type 1 diabetes experienced ≥ 3‐step DR progression [14], and after 3.9 years, the cumulative incidence of microalbuminuria was 11% [15]. Collectively, these observations illustrate a clear temporal gradient: prolonged exposure to hyperglycemia substantially increases the risk of vascular complications, with early manifestations emerging during adolescence and setting the stage for more advanced complications in young adulthood.

## 3. Risk Factors for Complications

Severe risk factors are implicated in the development of vascular complications, and they can be modifiable (hyperglycemia, hypertension, dyslipidemia, obesity, smoking) and non‐modifiable (diabetes duration, puberty, genetic predisposition, ethnicity, socioeconomic status) [16–18] (Figure 1).

### 3.1. Hyperglycemia

Hyperglycemia plays a pivotal role in the pathogenesis and progression of vascular complications. The DCCT/EDIC studies provided robust evidence for the association between hyperglycemia and the risk of developing neuropathy, DR, and DKD [19]. Achieving more stringent glycemic targets with intensive insulin therapy during the DCCT reduced the development and progression of DR by 76% and 54%, respectively, the incidence of moderately increased albuminuria by 39% and severely increased albuminuria by 54%, and the incidence of neuropathy by 60%. However, the generalizability of the findings warrants consideration, as DCCT primarily included participants at relatively low risk, excluding those with hypertension, hypercholesterolemia, albuminuria, or more than minimal retinopathy at baseline [19]. The outcomes for participants aged 13 to 17 years, although based on a relatively small sample size (*n* = 195), were consistent with those from the entire study population [20]. The DCCT showed that the relationship between HbA1c and complications is continuous but nonlinear. Although levels below 53 mmol/mol (7%) the risk is low risk, no HbA1c threshold could be identified, below which there was no risk for the development or progression of vascular complications [21].

More recent studies have confirmed the strong association between hyperglycemia, primarily assessed by HbA1c, and microvascular complications, as well as CVD risk, and stressed the importance of attaining good glycemic targets soon after diagnosis to prevent long‐term complications. Miller et al. showed that, after adjusting for baseline covariates, a high HbA1c trajectory was associated with a fivefold higher hazard of major adverse cardiovascular events versus a low trajectory (HR 5.10 [95% CI 2.94–8.86]). However, prior treatment strategies, baseline CVD exclusion (survivor/competing‐risk bias), and a 98% non‐Hispanic White cohort limit generalizability [19]. Bebu et al., using Cox proportional hazards models within the extended DCCT/EDIC cohort, demonstrated that each 1% increase in mean HbA1c confers a risk equivalent to 5.6 additional years of Type 1 diabetes duration for any CVD (95% CI 2.3–9); 18 additional Type 1 diabetes years for reduced glomerular filtration rate (GFR) /end stage renal disease (ESRD) (95% CI 4.3–31.7); 6.4 Type 1 diabetes years for PDR (95% CI 5.3–7.4) [16, 22–24].

### 3.2. Assessing Hyperglycemia: HbA1c and Beyond

HbA1c provides an objective estimate of average glycemic exposure over the previous 8 to 12 weeks and has been considered for a long time as the gold standard for monitoring glycemic control and guiding clinical decisions. However, HbA1c does not accurately capture blood glucose fluctuations and is influenced by several variables that are independent of blood glucose levels, such as age, ethnicity, pregnancy, anemia, nutritional deficiencies, and medication [25].

The use of continuous glucose monitoring (CGM) technology has enabled the assessment of short‐term fluctuations in blood glucose levels and to track them over time, identifying recurrent glucose patterns and facilitating the implementation of personalized therapeutic interventions [26].

Emerging evidence supports a strong correlation between CGM‐derived metrics and diabetes‐related complications, underlining their role in improving long‐term health outcomes in clinical practice. In a systematic review, Yapanis et al. demonstrated that markers of glucose variability (GV), such as mean amplitude of glycemic excursions and standard deviation of blood glucose levels, were consistently associated with microvascular outcomes. In addition, time in range (TIR) was independently linked to microvascular and macrovascular complications, including cardiovascular mortality and all‐cause mortality. Although less frequently studied, the coefficient of variation (CV) also showed a robust, independent association with complication risk after adjusting for HbA1c [27].

A retrospective cross‐sectional study in adults with Type 1 diabetes reported an inverse correlation between TIR and time in tight range (TITR) (the proportion of time spent with blood glucose levels between 3.9 and 7.8 mmol/L) with microvascular complications and cerebrovascular events. Importantly, these links were independent of HbA1c: per 10% higher TITR, odds were lower for any microvascular complication (OR 0.867, 95% CI 0.762–0.988), DR (OR 0.837, CI 0.731–0.959), background retinopathy (OR 0.831, CI 0.705–0.979), and cerebrovascular accident (OR 0.619, CI 0.426–0.899) [28]. Recent data for the pediatric population with Type 1 diabetes indicate that in both sexes, CGM hypoglycemia metrics and GV were positively correlated with body mass index (BMI), while low‐density lipoprotein cholesterol (LDL‐C) correlated positively with mean glucose and hyperglycemia metrics. LDL‐C was inversely correlated with TIR; specifically, TIR < 70% was linked to LDL‐C > 100 mg/dL (OR 3.2 in males, 2.1 in females), and in girls, CV > 36% was associated with overweight (OR 2.1) [29]. These findings suggest that CGM metrics may play an important role in cardiovascular risk assessment in children and adolescents.

### 3.3. Other Cardiometabolic Risk Factors

Type 1 diabetes, particularly when recommended glycemic targets are not achieved, is considered an independent cardiovascular risk factor [30]. Therefore, it is essential to screen young people with Type 1 diabetes for other cardiometabolic risk factors (Figure 1) [31].

Data from the Diabetes Prospective Follow‐up (DPV) registry indicated that 53% of young people with Type 1 diabetes had at least one cardiovascular risk factor, with prevalence rising with age and disease duration. The most common modifiable risk factors were hyperglycemia, overweight/obesity, and hypercholesterolemia [32]. Similarly, in the international SWEET registry, 41% of children and adolescents had one or more cardiovascular risk factors, and 10% had two or more. Common findings included LDL‐C > 2.6 mmol/L (100 mg/dL) (35%), BMI‐standard deviation score > +2 (26%), and systolic blood pressure (BP) > 90th percentile (17%). Factors associated with a higher risk of cardiovascular risk factors were female sex, HbA1c, and older age (> 10 years) [33]. The SEARCH study reported that 7% of youth with Type 1 diabetes had two or more cardiovascular risk factors and 1.7% had three or more. In these individuals, 26% of participants were overweight, 14% obese, 13% hypertensive, and 29% had dyslipidemia [32].

Other cardiovascular risk factors, such as smoking, are also common among youth with Type 1 diabetes, with percentages of 2.7%, 17.1%, and 34% among youth aged 10–14, 15–19, and > 20 years, respectively [34]. A high prevalence of overweight and obesity, which are traditionally associated with Type 2 diabetes, has been widely documented among youth with Type 1 diabetes. Registry data from the United States, Austria, and Germany showed a prevalence of overweight and obesity of 24% and 12%, respectively [35].

### 3.4. DKD

DKD has been identified as a significant risk factor for CVD events and mortality as well as all‐cause mortality in individuals with Type 1 diabetes. The association with CVD increases with the degree of albuminuria and renal function impairment [36].

Glomerular hyperfiltration has been associated with increased cardiovascular risk, even in individuals without diabetes, likely due to renin–angiotensin–aldosterone system activation and vascular dysfunction [37].

Urinary albumin‐to‐creatinine ratio (ACR) has emerged as a predictor of CVD outcomes in the general population as well as in individuals with diabetes or hypertension [38]. Even mild increases in albuminuria, within the normal range, are associated with increased risk of progression to moderately and severely increased albuminuria [39, 40]. In the AdDIT study, adolescents in the highest tertile of ACR had higher pulse wave velocity, non‐HDL‐C levels, apolipoprotein B/A1 ratio, and reduced levels of cystatin C and creatinine, than those with lower ACR levels [41]. In adjusted Cox models, being in the high‐ACR tertile conferred approximately fourfold higher risk of incident microalbuminuria (HR 4.29, 95% CI 2.08–8.85), and each 1% higher HbA1c increased the risk by 37% (HR 1.37, CI 1.10–1.72). They also exhibited greater progression in carotid intima‐media thickness (cIMT) and systolic BP, alongside larger shifts in estimated GFR [15].

### 3.5. Sex‐Related Differences

The prevalence of cardiovascular risk factors in youth with Type 1 diabetes also shows notable sex‐related differences. During adolescence, girls tend to have higher HbA1c, BMI, trunk fat, and prevalence of dyslipidemia than boys [22, 32]. In contrast, boys tend to have higher rates of smoking and high BP [32].

In adulthood, women have a higher relative risk of CVD than men, exhibiting a 40% excess of all‐cause mortality and twice the risk of lethal and nonlethal CVD compared with males [42].

The higher cardiovascular risk in women can be attributed to the complex interplay of genetic, hormonal, socioeconomic, and behavioral factors that contribute to the progressive loss of the well‐established cardioprotective effect of estrogens in premenopausal women without diabetes [43, 44]. Factors contributing to this process may include endocrine influences, such as hormonal fluctuations throughout the lifespan, which affect GV; psychological factors, including challenges in adaptation, disease acceptance, and a higher prevalence of depression, which may be more commonly observed in women with Type 1 diabetes than in men [44] and iatrogenic factors, such as the under‐treatment of cardiovascular risk factors, including dyslipidemia and hypertension, in women compared with men with Type 1 diabetes, as highlighted by several studies [45, 46].

However, data on sex‐related differences in microvascular complications are less consistent. In the SEARCH study, female sex (HR 5.23, 95% CI 1.15–23.91), older age at diagnosis (HR 1.27, 95% CI 1.10–1.48), higher HbA1c (HR 1.41 per 1% increase, 95% CI 1.18–1.68), and elevated mean arterial pressure were identified as predictors of microvascular complications. Although female sex emerged as a strong risk factor, the breadth of the confidence interval calls for caution in interpreting the robustness of this association [12]. A meta‐analysis of 10 studies including over 5 million participants indicated that diabetes confers a broadly comparable risk of chronic kidney disease in women and men (adjusted relative risk [RR] 3.34 vs. 2.84 [1.73–4.68]; women‐to‐men RRR 1.14 [0.97–1.34]). However, the risk of progression to ESRD appeared substantially higher in women (women‐to‐men RR 1.38 [1.22–1.55]; *I* ^2^ = 38*%*), suggesting that sex‐specific factors may influence susceptibility in the later stages of kidney disease [47]. Furthermore, a recent meta‐analysis highlighted that female adolescents are at increased risk of hyperfiltration, and normoalbuminuric phenotype of DKD appears to be more prevalent in women [48].

## 4. The Role of Predictive Models and Personalized Medicine

Cardiovascular risk in individuals with Type 1 diabetes varies widely. Precision prognostics could help identify individuals at greater risk. This would enable physicians to provide tailored information, raise their awareness about potential complications, and adjust the intensity of treatment according to the individual’s risk level [49]. However, the development of predictive models is complex and requires the integration of multiple data such as demographic, clinical, and biochemical parameters, along with lifestyle information and medications. This complexity is further increased when the outcomes are represented by diabetes complications because of the intricate interactions that exist between them [50].

Several validated models are available for predicting vascular complications in individuals with Type 2 diabetes. Among these, the RECODe (Risk Equations for Complications of Type 2 Diabetes) model demonstrates robust performance in external validation studies, with C‐statistics ranging from 0.72 for stroke to 0.87 for cardiovascular death. Calibration has also been reported as generally good, with slopes close to unity (approximately 0.94–1.10 for major cardiovascular outcomes), and comparable results for microvascular complications, where slopes ranged from 0.62 to 1.07. However, its generalizability to youth with Type 1 diabetes is limited, and diverse populations remain underrepresented [51].

A recent systematic review including over 73,000 individuals with Type 1 diabetes evaluated 12 cardiovascular risk prediction models and found that models specifically developed for Type 1 diabetes generally outperformed those created for Type 2 diabetes, both in terms of discrimination (pooled C‐statistic: 0.81) and calibration. Among these, the Swedish National Diabetes Register (NDR) Type 1 Diabetes Cardiovascular Risk Model, the EURODIAB Prospective Complications Study Risk Model, and the Steno Type 1 Risk Engine (ST1RE) showed particularly strong performance, with C‐statistics ranging from 0.80 to 0.82, consistent with findings from their respective original studies [52]. The NDR model reported a C‐statistic of 0.83 in derivation and 0.80 in external validation, with good calibration (predicted/observed risk ratio 0.94) [53]. EURODIAB Prospective Complications Study Risk Model showed C‐statistics of 0.74 in the derivation cohort and 0.79–0.82 in external validation cohorts, with good overall calibration reported [54]. ST1RE similarly demonstrated good calibration (Hosmer–Lemeshow *p* = 0.136 at 5 years, *p* = 0.052 at 10 years) and excellent discrimination in both derivation (*C* = 0.826 and 0.818 at 5 and 10 years) and external validation, including in ethnically diverse cohorts and in individuals under 40 years of age (*C* = 0.912 and 0.916 at 5 and 10 years, respectively, with good calibration) [55].

These models have been developed and validated using large, well‐characterized cohorts and align with findings from their respective original studies. While each includes a slightly different set of predictors—such as diabetes duration, HbA1c, renal function, and BP—they all showed good performance in predicting cardiovascular outcomes.

However, only a small proportion of these have been developed and validated in populations with Type 1 diabetes [52]. These models incorporate a variety of demographic data, including age, sex, diabetes duration, and ethnicity. They also consider clinical parameters, such as BP, BMI, along with smoking habits, lifestyle, and history of previous CVD, medications, and biomarkers, including lipids, HbA1c, and renal function.

Current research focuses on identifying new biomarkers related to the genome, transcriptome, proteome, or metabolome that may facilitate earlier identification of complications and risk stratification in people with Type 1 diabetes. The introduction of these novel biomarkers could complement existing risk factors and extend the predictive capacity for diabetes complications by contributing to the development of risk scores [49]. Additionally, incorporating subtle changes—such as high‐normal ACR values—could enhance early risk detection [14].

## 5. Early Detection and Screening

The latest guidelines from the International Society for Pediatric and Adolescent Diabetes (ISPAD) advocate for the early initiation of screening for predisposing factors and the early detection of complications in youth with diabetes (Table 1) [17].

**Table 1:** Recommendations for screening diabetes complications in children and adolescents from the International Society for Pediatric and Adolescent Diabetes.

**Complication**	**Screening methods**	**Start and frequency**	**Abnormal results**
Kidney disease	Urinary albumin/creatinine ratio (ideally on first morning urine sample)	Start: age 11 years with 2–5 years diabetes duration Frequency: annually	Two out of three samples show increased albuminuria

Retinopathy	Fundal photography or mydriatic ophthalmoscopy	Start: age 11 years with 2–5 years diabetes duration Frequency: every 2–3 years (or more often if abnormal findings)	Vision‐threatening retinopathy Diabetic macular edema with vision loss

Neuropathy	History Physical examination Clinical tests	Start: age 11 years with 2–5 years diabetes duration Frequency: annually	Neuropathic pain

Cardiovascular risk factors	Blood pressure Lipid profile Discourage smoking Promote healthy diet and physical activity	At every visit; except for lipids—to be checked at diagnosis and then from age 11 years every 3 years	Blood pressure > 90^th^ percentile LDL‐cholesterol > 2.6 mmol/L

Since vascular complications often arise from a subclinical disease starting in adolescence, early evaluation of modifiable risk factors can reduce long‐term complications. ISPAD recommends commencing screening for any vascular complications at the time of diagnosis in individuals with Type 2 diabetes. In contrast, for individuals with Type 1 diabetes, it recommends initiating screening for microvascular complications at age 11 or puberty (whichever comes first) with 2‐5 years of disease.

For the prevention of CVD, guidelines recommend measuring lipid levels and BP. BP should be assessed at each visit, or at least once a year, whereas screening for dyslipidemia is advised at the diagnosis of Type 1 diabetes, with subsequent reassessment of lipid profiles at 11 years with 2–5 years of disease. Subsequent screening can be performed every 3 years if the initial results are normal. For the detection of albuminuria, ISPAD recommends that initial screening include measurement of urine ACR, ideally on a first‐morning sample, on an annual basis. If pathological values are identified (≥ 30 mg/g or 3 mg/mmol), two additional samples should be collected early in the morning and at least two out of three pathological samples over a 3‐ to 6‐month period are required to confirm persistent albuminuria. Regarding DR, ISPAD guidelines recommend performing screening by fundus photography or mydriasis ophthalmoscopy, to be repeated every 2–3 years in individuals who do not show any signs of retinal involvement or more often if there are abnormal findings. Regarding neuropathy, ISPAD guidelines recommend an annual clinical evaluation with history, physical examination, and clinical tests, for all individuals with diabetes (Table 1).

## 6. Treatment

**Figure 2:**
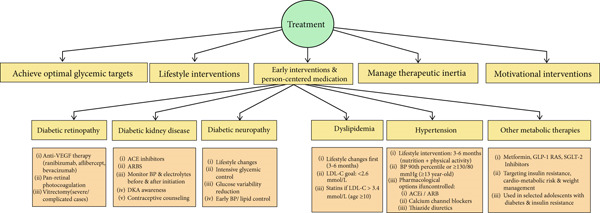
Managing diabetes complications in adolescents.

Timely therapeutic interventions are essential for adolescents with Type 1 diabetes when early manifestations of vascular complications are detected to reverse early abnormalities and prevent progression (Figure 2). The early management of complications is based on three pillars: achieving recommended glycemic targets, promoting healthy lifestyle modifications, and considering additional pharmacological therapies when indicated [17] (Figure 2).

## 7. Achieving Optimal Glycemic Targets

Adolescents face unique physiological and psychological challenges that distinguish them from both younger children and adults. Achieving recommended glycemic targets during adolescence is challenging, and a substantial increase in HbA1c levels during this period of life is commonly seen, leading to an increased risk of diabetic ketoacidosis (DKA) [56, 57]. A tailored approach is essential for managing the physiological demands of Type 1 diabetes, including the need for frequent dose adjustments, while also supporting psychological well‐being. This involves addressing a range of behavioral responses to treatment, from impulsiveness and doubt to the challenges of managing exhaustion and anxiety that often accompany the management of chronic conditions [58]. A multidisciplinary pediatric diabetes team should be readily available to support and educate adolescents and their families on the importance of preventing complications [59].

Maintaining optimal glycemic levels during adolescence and early adulthood is crucial for reducing the risk of vascular complications. Both ISPAD and the American Diabetes Association (ADA) have consistently revised their recommendations on optimal glycemic targets over the past two decades, with the objective of ensuring favorable health outcomes for young people with Type 1 diabetes [60, 61]. The recommended 24‐hour target glucose levels should be between 3.9 and 10 mmol/L, with at least 70% of the time within this range. For HbA1c, a target of less than 7.0% (53 mmol/mol) or even lower (< 6.5%, 48 mmol/mol) is recommended by most national and international guidelines [60, 61].

However, it is essential to emphasize that the achievement of stringent glycemic targets, as reflected by HbA1c levels, should always balance the critical need for hypoglycemia prevention. Indeed, beyond its immediate risks, hypoglycemia represents a long‐term factor contributing to the development of CVD in people with Type 1 diabetes [62, 63].

## 8. Lifestyle Interventions

ISPAD and ADA guidelines suggest a series of health objectives to improve the long‐term outcomes of young people with diabetes (Table 2). It is recommended that HbA1c levels be maintained below 48–53 mmol/mol (6.5%–7%), BMI below the 85th percentile, systolic, and/or diastolic BP below the 90th percentile for age and sex, LDL‐C below 2.6 mmol/L (100 mg/dL), not smoking, reducing sedentary behaviors and following a healthy diet [17]. The health targets set by the ISPAD guidelines for young people with diabetes align with the guidance provided in 2022 by the American Heart Association (AHA) regarding the eight essential determinants (Life’s Essential 8) for achieving good cardiovascular health, which also include healthy sleep [64]. Evidence suggests that the greater the number of healthy cardiovascular metrics achieved, the lower the risk of CVD, CVD‐related mortality, and all‐cause mortality. In adults with Type 1 diabetes, achieving recommended cardiovascular health metrics has been associated with better cardiovascular outcomes [65, 66]. In a study conducted on 297 adolescents with Type 1 diabetes, those who achieved ideal values for at least four of the cardiovascular targets set by ISPAD and ADA had lower arterial stiffness and hyperfiltration [67].

**Table 2:** Behavioral recommendations per age group—ISPAD and ADA Guidelines recommendations.

**Behavior**	**Age group**	**Recommendation**
Healthy eating	< 11 years	Focus on balanced diet, involve family in meal planning
	11–18 years	Encourage healthy eating habits, monitor intake

Physical activity	< 11 years	At least 60 min of physical activity daily
	11–18 years	Encourage regular physical activity, reduce sedentary time

Blood glucose monitoring	< 11 years	Emphasize regular monitoring, involve parents
	11–18 years	Encourage self‐monitoring, educate on target ranges

Medication adherence	< 11 years	Involve parents, use reminders
	11 ‐ 18 years	Educate about importance, use reminders or apps

Stress management	< 11 years	Teach coping skills, involve school support
	11–18 years	Encourage stress‐relief activities, provide mental health resources

Screen time	< 11 years	Limit screen time to 1–2 h/day
	11–18 years	Encourage alternative activities, set screen‐free times

Sleep hygiene	< 11 years	Ensure 9–12 h of sleep per night
	11–18 years	Ensure 8–10 h of sleep per night, maintain regular sleep schedule

Alcohol and substance use	< 11 years	Educate on risks, discourage use
	11–18 years	Strongly discourage use, provide resources for prevention

Tobacco use	< 11 years	Educate on risks, discourage use
	11–18 years	Strongly discourage use, provide resources for cessation

## 9. Early Interventions—Pharmacological and Surgical Treatment Options (Figure 2)

### 9.1. DR

Vision‐threatening DR can be treated with pan‐retinal laser photocoagulation (PRP) or intravitreal anti‐vascular endothelial growth factor (VEGF) therapy injections (e.g., ranibizumab, aflibercept, bevacizumab) [17]. PRP creates discrete retinal burns in the peripheral retinal areas while sparing the macula. While PRP halts disease progression, anti‐VEGF therapy has shown superior short‐term visual acuity outcomes and reduced diabetic macular edema. However, anti‐VEGF treatment requires frequent injections—often monthly in the first year—and carries a small risk of ocular infection. Long‐term data from The Diabetic Retinopathy Clinical Research Network Protocol S suggest comparable visual acuity between the two approaches, with anti‐VEGF offering better preservation of visual fields and lower incidence of diabetic macular edema. Surgical options like vitrectomy are reserved for cases with retinal detachment, vitreous hemorrhage, or severe fibrosis [17]. Most of the data on these interventions come from adults with Type 1 diabetes, with limited experience in youth.

### 9.2. Dyslipidemia and Hypertension

A three‐ to six‐month nutritional and physical activity intervention should be offered to adolescents with Type 1 diabetes and dyslipidemia (defined as a fasting serum LDL‐C level > 2.6 mmol/L) or elevated BP (defined as BP ≥ 90th percentile for age, sex, and height, or from the age of 13 years as BP between 120 and 129/80 mmHg). The same approach applies to those with hypertension (BP equal to or above the 95th percentile for age, sex, and height, or from the age of 13 years, with systolic BP ≥ 130 and/or diastolic BP ≥ 80 mmHg) [17].

If BP remains elevated, the physician should consider initiating antihypertensive drug therapy with an angiotensin‐converting enzyme inhibitor (ACEi), an angiotensin II receptor blocker (ARB), a long‐acting calcium channel blocker, or a thiazide diuretic [17] If LDL‐C levels remain > 3.4 mmol/L despite lifestyle intervention, children over 10 years should be offered statin treatment, aiming for LDL‐C <2.6 mmol/L. Similarly, persistent hypertension warrants pharmacological treatment [17].

Lifestyle interventions can have beneficial effects in achieving better targets for lipids and BP in this age group, but additional therapeutic options are often necessary [68].

The AdDIT trial, a double‐blind, randomized, placebo‐controlled trial, demonstrated the efficacy of statin treatment in reducing serum levels of total, LDL‐C, and non‐HDL‐C and in improving the Apolipoprotein B/A1 ratio in adolescents with Type 1 diabetes [69]. The trial also demonstrated the favorable impact of ACEi on endothelial dysfunction, evidenced by their protective effect on FMD [70]. In addition, the safety of statins and ACEi in adolescents was demonstrated. No serious side effects were detected, and there were no concerns about liver function associated with statin use. Similarly, the use of ACEi did not elicit any notable adverse reactions, with the exception of postural hypotension, which was successfully addressed by reducing the dosage [69].

### 9.3. Albuminuria

The use of ACEi or ARBs is recommended for the treatment of hypertension and albuminuria in adolescents with Type 1 diabetes. Still, the evidence for their use in the presence of the sole albuminuria is less robust in this population than in adults [71]. The AdDIT study did not demonstrate a statistically significant effect of ACEi on the area under the curve of ACR. However, there was a 43% reduced risk of developing moderately increased albuminuria compared with placebo [69] Furthermore, concerns have been raised regarding the potential for these drugs to be used in adolescents for decades, given the possibility of adverse effects and the risk of teratogenicity [17] A series of measures can be taken by physicians to address the concerns. The safe administration of ACEi in adolescents would include the monitoring of BP and electrolyte levels prior to the initiation of therapy and for a period of 2 to 4 weeks following the commencement of treatment and annually thereafter. To address concerns regarding hypotension, a test dose could be given in the hospital setting with pre‐ and post‐BP monitoring, prior to the commencement of regular treatment. ACEi/ARBs should be discontinued during episodes of dehydration and DKA. It is also recommended that contraception counseling be provided to postpubertal females because of the potential teratogenic effects of treatment [17].

### 9.4. Other Potential Therapeutic Strategies

Insulin resistance can be a common finding among adolescents with Type 1 diabetes, particularly in the context of obesity or overweight, and represents a significant cardiovascular risk factor [72]. Consequently, a few clinical trials have assessed the potential effect of insulin‐sensitizers medications in youth with Type 1 diabetes. Metformin is an oral glucose‐lowering agent that is commonly used in the treatment of Type 2 diabetes. It exerts its effect by increasing the uptake of glucose by tissues, particularly muscle tissue, and reducing hepatic glucose secretion [73]. A few studies have assessed Metformin in youth with Type 1 diabetes and primarily shown effects on improving measures of adiposity, insulin sensitivity and vascular function measure [18, 74–77].

New therapeutic options, including GLP‐1 receptor agonists (GLP‐1 RAs) and SGLT‐2 inhibitors, are currently used in adults and in youth with Type 2 diabetes, but there are limited data in youth with Type 1 diabetes [78]. GLP‐1 RAs promote insulin secretion in individuals with residual *β*‐cells, slow gastric emptying, inhibit postprandial glucagon release. They have been shown to be effective in Type 1 diabetes in reducing HbA1c, total daily dose, and weight. However, increased the risk of hypoglycemia and ketosis [79].

Although some studies suggest GLP1‐RAs may help preserve insulin secretion or improve metabolic markers, results remained inconsistent, and gastrointestinal adverse events were common. Some GLP‐1 RAs, such as exenatide and albiglutide, have shown limited or no benefit in Type 1 diabetes.

While GLP‐1 RAs have demonstrated metabolic benefits in adults with Type 1 diabetes, their use in children and adolescents remains insufficiently supported by robust evidence. Notably, the off‐label use of GLP‐1 RAs among youth with Type 1 diabetes, particularly those with obesity, has increased in recent years, emphasizing the need for rigorous pediatric studies to inform practice [80].

Sodium–glucose linked transporter‐2 (SGLT2) inhibitors exert their effect independently of insulin by reducing renal tubular glucose reabsorption. Clinical trials demonstrated improvements in HbA1c, insulin doses, BP, and weight in people with Type 1 diabetes, although several studies have documented an increased risk of DKA [80]. A randomized controlled trial in adolescents and young adults with Type 1 diabetes demonstrated that dapagliflozin, when used as an adjunct to hybrid closed‐loop systems, significantly improved TIR, nocturnal glycemic profile, and reduced insulin total dose, without increasing risk of hypoglycemia or DKA [81]. However, this study did not assess the long‐term effect of dapagliflozin on complications. Sotagliflozin, a dual SGLT1/2 inhibitor, may offer additional benefits in postprandial glucose control and reduced risk of hypoglycemia, with a more favorable safety profile in individuals with higher BMI [80]. More recently, the ATTEMPT trial [Adolescent Type 1 Diabetes Treatment with SGLT2i for hyperglycEMia & hyperfiltration Trial] evaluated dapagliflozin in 98 adolescents and young adults with Type 1 diabetes and demonstrated significant reductions in measured glomerular filtration rate (mean difference –8.8 mL/min/1.73 m^2^; 95% CI –12.7 to –4.8) together with improvements in HbA1c (–0.47%; 95% CI –0.66 to –0.28), Time in Range (+9 percentage points; 95% CI +3.8 to +14.3), and body weight (–2.8 kg; 95% CI –3.7 to –2.0). Despite the trial’s relatively short 22‐week duration, modest sample size, and inclusion of participants with preserved renal function, the findings provide encouraging guidance for future long‐term studies, as DKA incidence remained low with structured ketone monitoring and risk mitigation strategies, supporting the potential metabolic and renal benefits of dapagliflozin in this population [82].

### 9.5. Therapeutic Inertia

Although CVD remains the leading cause of death in people living with diabetes, key risk factors such as hypertension and dyslipidemia are often underdiagnosed and largely undertreated in youth with diabetes [83]. The prescription of antihypertensive medications and statins is frequently delayed in this population and lifestyle interventions are primarily recommended, despite healthcare professionals are doubtful about their effectiveness [84].

Therapeutic inertia, defined as the lack of timely adjustment to therapy when a patient’s treatment goals are not met, arises from several factors, including a lack of support for people with diabetes in achieving and maintaining their health goals, along with insufficient motivation and confidence. From the perspective of healthcare professionals, additional challenges include a perceived shortage of educational material, limited time to devote to diabetes complications, and a lack of familiarity and experience in prescribing antihypertensive and lipid‐lowering medications for this age group.

There is also a general lack of parental confidence in their children’s treatment for dyslipidemia and hypertension, which poses a significant barrier to achieving cardiovascular health goals. Furthermore, while adolescents may be receptive to pharmacological interventions for cardiovascular risk factor management, they are reluctant to endure the inconvenience and discomfort of taking daily medication in addition to insulin therapy [85].

### 9.6. Motivational Interventions

Healthcare professionals should discuss with adolescents with Type 1 diabetes the risk of complications and their potential impact on long‐term health outcomes. This information can motivate them to achieve and maintain good glycemic targets and a healthy lifestyle. Providing structured and age‐appropriate information can enhance motivation and engagement; however, this process is often challenged by the emotional sensitivity and limited knowledge. Qualitative studies indicate that adolescents frequently obtain fragmented or inaccurate information from nonmedical sources. In contrast, parents tend to have a broader understanding, but generally are unsure how to approach the topic [86].

Parents should be empowered to collaborate with the diabetes team to determine the most appropriate approach for educating their children about the long‐term complications of diabetes. It is recommended that families receive basic information about vascular complications at the onset of diabetes, with content gradually expanded over time according to their interest and readiness [87]. Targeted behavioral guidance can also support this approach. For instance, structured physical activity (≥ 60 min/day) and parental involvement in medication adherence and blood glucose monitoring. In contrast, adolescents aged 11–18 years require greater autonomy in diabetes management, supported by digital tools (e.g., reminder apps), peer‐appropriate education on substance use, and access to mental health resources (Table 2) [85].

These strategies reinforce the need for greater awareness of long‐term risks and the improvement of age‐appropriate, collaborative communication frameworks.

## 10. Conclusions

Despite significant advances in the management of Type 1 diabetes over recent decades, vascular complications remain a major burden for individuals diagnosed at a young age. For health care professionals caring for young people with Type 1 diabetes, the primary objective should be early identification of those at risk, followed by timely preventive and therapeutic interventions. Raising awareness of complications among young people and their families, along with addressing therapeutic inertia, is essential to this approach. Efforts to improve vascular outcomes should begin during adolescence, shifting from reactive management of established conditions to proactive prevention strategies. Ongoing work is needed to develop more effective screening tools and evidence‐based treatment options. Future research should prioritize addressing these critical gaps by developing and validating advanced predictive models that accurately identify high‐risk individuals, support early diagnosis, and guide personalized intervention strategies to reduce long‐term complications and improve health outcomes in youth with Type 1 diabetes.

**Nomenclature**
AdDIT: Adolescent Type 1 Diabetes cardiorenal Intervention Trial
ACR: albumin‐to‐creatinine ratio
ADA: American Diabetes Association
ACEi: angiotensin converting enzyme inhibitors
ARB: angiotensin II receptor blocker
ATTEMPT trial: Adolescent Type 1 Diabetes Treatment with SGLT2i for hyperglycEMia & hyperfiltration Trial
BP: blood pressure
BMI: body mass index
CVD: cardiovascular disease
CV: coefficient of variation
CGM: continuous glucose monitoring
CI: confidence interval
DCCT: Diabetes Control and Complications Trial
DKA: diabetic ketoacidosis
DKD: diabetic kidney disease
DR: diabetic retinopathy
DPV: Diabetes Prospective Follow‐up
EDIC: Epidemiology of Diabetes Interventions and Complications
ESRD: end stage renal disease
FMD: flow‐mediated dilatation
GFR: glomerular filtration rate
GLP1: glucagon‐like peptide 1
GLP1‐RA: glucagon‐like peptide‐1 receptor agonists
GV: glucose variability
HbA1c: glycated hemoglobin
HDL‐C: high‐density lipoprotein cholesterol
HCL: hybrid closed‐loop
HT: hazard ratio
ISPAD: International Society for Pediatric and Adolescent Diabetes
LDL‐C: low‐density lipoprotein cholesterol
NDR: National Diabetes Register
PAR: pan‐retinal laser photocoagulation
RECODe: Risk Equations for Complications of Type 2 Diabetes
RR: relative risk
SGLT1/2: sodium‐glucose linked transporter‐1/2
ST1RE: Steno Type 1 Risk Engine
TIR: time in range
TITR: time in tight range
TODAY: Treatment Options for Type 2 Diabetes in Adolescents and Youth
VEGF: vascular endothelial growth factor

## Disclosure

The authors have nothing to report.

## Conflicts of Interest

The authors declare no conflicts of interest.

## Author Contributions

Beatrice Prampolini performed the literature review and wrote the initial draft of the manuscript. Cansu Ceren Eryılmaz performed the literature review and edited the manuscript. M. Loredana Marcovecchio critically reviewed and edited the manuscript. All authors reviewed and approved the final draft.

## Funding

No funding was received for this manuscript.

## Data Availability

This is a review of the literature, and no research data are reported.
